# Nature Immersion in an Extreme Environment: Hiroshima Survivors’ Personal Emergence Following Their Atomic Bomb Experience

**DOI:** 10.3390/ijerph192315894

**Published:** 2022-11-29

**Authors:** Misako Nagata, Mio Ito, Ryutaro Takahashi, Chie Nishimura, Patricia Liehr

**Affiliations:** 1 Research Institute for Next-Generation Nursing Education, 3-26-1-702 Hongo, Bunkyo-ku, Tokyo 113-0033, Japan; 2 Graduate School of Health Sciences, Gunma University, 3-39-22 Showa-machi, Maebashi 371-8514, Japan; 3 Tokyo Metropolitan Institute of Gerontology, 35-2 Sakae-cho, Itabashi-ku, Tokyo 173-0015, Japan; 4 St. Luke’s Clinic-Ala Moana, 1141 Kapiolani BLVD #2000, Honolulu, HI 96814, USA; 5 Christine E. Lynn College of Nursing, Florida Atlantic University, 777 Glades Road, Boca Raton, FL 33431, USA

**Keywords:** nature immersion, green space, natural space, self-healing, healing, extreme environments, radioactive nuclear energy, Hiroshima atomic-bombing survivors, A-bombing survivors

## Abstract

Introduction: Nature immersion is defined as multidimensional connecting with earthy materials to generate personal emergence. Personal emergence is an embodied healing force observable via synchronization of bodily rhythms. Research has revealed positive effects of green space for healing. However, little is known about healing of survivors in the space impacted with radioactive nuclear energies. Purpose: To use the theory of nature immersion to guide exploration of the concepts of *connecting with earthy materials*, *personal emergence* and *space-time expansion* in a sample of people who had experienced the catastrophic nature upheaval of the Hiroshima bombings on 6 August 1945. Method: A descriptive exploratory design with directed content analysis was used with existing qualitative data consisting of 29 Hiroshima atomic-bombing survivors’ description of their experience. Results: Self-healing empirically manifested through 23 survivors’ connection with earthy materials. There was *synchrony* between recuperating natural space and healing of survivors. Conclusions: Synchrony, as a dimension of human connection with nature, transcended the disharmony of bombing upheaval. Although further exploration is necessary, these findings serve as evidence about the essence of healing as related to nature for those in extreme environments.

## 1. Introduction

Nature immersion is connectedness between earthy materials and a person that generates personal emergence, an embodied healing force [1,2,3]. Humans’ natural affinity with elements of green space, considered a phenomenon of nature immersion, as known through the Biophilia hypothesis [4], appears to be an inherent drive toward personal emergence. It was said that the emergence of the flowers in the aftermath of atomic fallout that created a wasteland in Hiroshima encouraged many Hiroshima survivors to believe in a future and to pursue their own future. One plant, *Canna indica*, flowered within one kilometer of the atomic bomb epicenter in the autumn of 1945, only a few months after the fall of the bomb [5] (p. 83). The flower’s monochrome picture is presented at the Hiroshima Peace Memorial Museum today. This flower’s emergence was a harbinger, suggestive of a fundamental impetus generated from within an individual living system for streaming new life. Personal emergence indeed is life force that self-organizes in synchrony with environments. New ecological relationships or rhythms emerge for a person as expressions of personal emergence. According to a *nature immersion* model, originally developed in 2018, a person generates his or her own personal emergence through connecting with earthy materials [1]. There must have been some levels of connection or synchrony of life force between Hiroshima atomic-bomb survivors and earthy materials, which enabled survivors to exert their own personal emergence. Personal emergence cannot be forced but flows freely, creatively, and unpredictably, creating something new rather than something that previously existing [6], like a flower that cannot be forced to bloom but blooms by flow of ecological synergy with environmental fields of energy. 

The purpose of this study was to use the theory of nature immersion to guide exploration of the concepts of *connecting with earthy materials*, *personal emergence,* and *space-time expansion* in a sample of people who had experienced the catastrophic nature upheaval of the Hiroshima bombings on 6 August 1945. Using an existing data set collected decades after the bombing, a secondary analysis was intended to enhance understanding of how individuals “touched” nature in spite of toxic devastation and personally emerged in spite of seemingly insurmountable odds. 

### 1.1. Literature Related to Nature Immersion

Original work on the idea of nature immersion resulted in a middle range theory of nature immersion. The theory proposes that nature immersion has the power to generate personal emergence, a healing force emerging from within a person who is connecting with earthy materials. This healing force is shaped with a third theory concept, space-time expansion [3]. According to the theory model [1,3], which was developed and refined from studying parental reports about the well-being of their urban children [2] while *being* and *doing* in nature, nature-based experiences can nurture child well-being even when there is a deficit of natural space. Furthermore, well-being progress is influenced by space timeframe factors such as closeness to and time spent in nature. Although important, this finding is not transferrable to those who are in extreme environments with both deficit and destruction of natural space, like the survivors of Hiroshima. Space-timeframe issues further complicate transferability when nature environments are toxic. These consideration led to further exploration of extant literature related to the human impact of nature and the secondary analysis described in the manuscript. 

A person immersed in nature experiences oscillatory rhythms both internally (e.g., heart, brain) and externally (e.g., Earth’s energy, such as solar and geo-electromagnetic fields). These oscillations manifest in environmental/emotional responses such as fluctuations in the between-beat heart rate. For instance, internally, buffeting of the sympathetic and parasympathetic nervous systems in the sinus-node cells of the heart continues in a healthy person as *auto-adjustment* [7]. Complexities of rhythmicity in oscillatory human patterns from cellular to cultural are fluctuating like a dance in a rhythmic medium of nature, the natural space. The dance or biodance [8] is the constant state of change and exchange with all the organic and inorganic parts of nature dynamically interacting at the atomic through cosmic level. Due to the complexities, the biodance surfaces in a nonlocal and nonlinear manner, not necessarily following from a unidirectional time series. 

One’s oscillatory nature entrains rhythmicity of another’s oscillatory nature into synchronization known as *psychophysiological coherence* [9] (p. 16). The state is known to occur with a synchronicity of brain (EEG)-heart rhythms (ECG) both within and between individuals [10,11]. This state of coherence provides the best functional mode in oscillatory bodily systems, wherein efficient utilization of energy arises [12]. Personal emergence, an embodied healing force, may generate through synchronization of one’s own internal and external rhythmic patterns. Any of the bodily rhythms of breathing, feeling, thinking, and healing [13] could be the manifestation of healing force. 

The phenomenon of healing is wholistic and unitary, being experienced within the complex and constant biodance [8]. Its unitary quality makes it very challenging for the researcher to study. Nevertheless, observation of nature immersion may provide one inroad from which the not-easily graspable biodance of nature immersion can be explored. 

There have been varied frameworks documenting the beneficial impacts of nature (green-space)-immersion on health [14,15,16]. Recently, the biodiversity-health framework [17] was introduced to describe mechanistic pathways by which biodiversity can be linked to health and well-being. The framework authors emphasize a distinction between nature (essentially non-human physical features that can be perceived by humans) and biodiversity (qualities of living organisms and ecosystems), making the case that biodiversity allows for a broader and more measurable perspective. The state of biodiversity could be both actual and perceived experiences, both of which could be measured [17]. Considering this secondary analysis of Hiroshima data, quantification of biodiversity would have been challenging given the extreme environment. Earthy materials of the green space were reportedly leveled into ashes [5]. Both entities of biodiversity and humans were in chaos in the extreme environment of Hiroshima, complicating exploration of causal relationships. 

The challenge of studying atomic bombing chaos was emphasized with the use of a linear model in the Life Span Study (LSS) of atomic bombing survivors in Hiroshima and Nagasaki. Critical variables composing the real world (*radiation hormesis*, *black rain exposures*, and *anthropogenic organic materials*), which were integral to the environment and powerful space timeframe factors, were not incorporated into the model, making use of the mechanistic linear model analysis controversial [18,19]: those real-world variables occurred irregularly, unpredictably, and with uncertainty in the chaos of Hiroshima fallout. Hormesis is a uniquely adaptive response of cells and organisms to a moderate stress, ranging from the low-dose beneficial stimulatory effects to the high-dose toxic inhibitory effects [20]. Radiation hormesis is a dose and dose-rate sensitive response of living systems from ionizing radiation [18]. Besides initial radiation from the blast, black rain, the major fallout, irregularly showered at least three times with irregular intervals and levels of radioactive dose/dose-rate each time, ensuing in unpredictable radiation hormesis for the survivors and other host entities [18,21]. Uncertain volatile components of anthropogenic organic materials (e.g., houses, buildings, human bodies, etc.) were the water-source of the black rain which was produced by the Hiroshima inferno [18,21]. Chaotic environments, like the one experienced at Hiroshima, are highly sensitive so that small variations in the system generate high unpredictability [22]. 

Chaos and complexities are the hallmark of non-linear mathematics: complex systems science [23]. Natural space is embedded with increasing entropy, a descriptor of disorder and a concept related to spatial-temporal processes, where order marches into disorder [24,25]. However, the chaotic environments, oscillating stably in a nonlinear manner, converge to a new equilibrium out of the disequilibrium within predictable boundaries [24,25]. The state of healing in this context of complexity is not just going back to the previous state of health, but a newly emerging order. For instance, observable resilience of toxic nature-survivors could be expression of a new emerging order of health: self-healing. The complexities of studying chaos have been explored in intricate systems of healthcare [26], cardiac events (e.g., arrhythmia [27]), and life-altering disaster [28]. Qualitative research offers a unique human science approach to investigation when non-linear processes predominate as in the case of the bombings of Hiroshima on 6 August 1945. 

### 1.2. Introduction to Qualitative Research Analysis

Traditional scientific research models, which rely on quantitative strategies inclusive of measurement, generalization, and predictability, are the defining core of medical science where causation and cure are paramount. However, a view of the world that seeks understanding through exploration and description rises as a meaningful complementary model; one that is distinguished by qualitative narrative approaches that embrace the complex contextual nature of human experience [29]. Inherent in the narrative approach of qualitative research are the ideas of science “with heart”, engaged listening and acceptance of unique patterns of knowing [30] as well as recognition of the positionality of the researcher [31]. The narrative evidence shared by Hiroshima atomic-bomb survivors was gathered by Japanese researchers interested in how health emerged over decades of survival. 

In this secondary analysis, the relationship between nature immersion, specifically radioactive nature and health, was addressed by an interdisciplinary, cross-cultural team of researchers. Marselle and colleagues [17] indicate that studies linking biodiversity (inclusive of nature) and human health demand interdisciplinary teams. Although these scholars [17] propose a predictive model and arrive at causal pathways that are inconsistent with the essence of chaotic circumstances, there is common ground between their ideas and that of narrative science in an appreciation for ways to grapple with the complexity. The qualitative data from Hiroshima survivors underwent rigorous directed content analysis., guided by the middle range theory of nature immersion. To assure qualitative analysis integrity, standards of methodological rigor (transferability; confirmability; credibility) critical to narrative science were implemented [32,33] as follows:

1. Transferability (parallel to external validity): Findings are more transferable if there is purposive sampling and detailed rich description, qualities addressed through recruitment of documented Hiroshima survivors and reporting of direct quotes that support the thematic findings.
2. Confirmability (parallel to objectivity): Findings are confirmable when another researcher can observe and audit activities to affirm plans for data preparation, collection, and analysis. In this instance, the researcher kept detailed records of interpretive thought processes and decisions which ensued from the processes, sharing records with parent study researchers for peer debriefing.
3. Credibility (parallel to internal validity): Findings are credible when investigated with other researchers to consider comparative analysis and triangulation of findings. The data collection process contributes to credibility when the participants’ narratives are honored with inquiry that ensues without researcher bias. In this study, all researchers used a structured data-gathering approach that called for non-judgmental authentic presence. The research team had worked together for more than a decade prior to data collection, allowing for trusted engagement. Engagement occurred throughout all phases of analysis including this secondary analysis.

While the evaluation of rigor differs for quantitative and qualitative studies, it is consistently an indicator of quality investigation. The rigor of this study beginning with the original parent study to the current secondary analysis was strengthened by attention to transferability, confirmability, and credibility.

## 2. Materials and Methods

This research utilized an existing data set that queried the human health experience of surviving the atomic bombing of Hiroshima [34]. The original data set was explored guided by three concepts of the nature immersion theory, connecting with earthy materials, personal emergence, and space-time expansion (Table 1). 

This analysis offers an opportunity to increase our understanding about: (1) personal emergence as an embodied healing force for people who had been immersed in radioactive nuclear energy; (2) connection with earthy materials that contributed to personal emergence during and since this catastrophic event; and (3) space-time expansion as an impactful health-related factor in toxic environments. The research questions were formulated corresponding to the pursuit of understanding: (1) What *personal emergence* (self-healing) was manifested among Hiroshima atomic bomb survivors from postwar onward to present time?; (2) How did Hiroshima survivors *connect with earthy materials* in the aftermath of the atomic bombing through which personal emergence generated; and (3) What *space-time factors* contributed to personal emergence? 

Sampling. Stories from Hiroshima atomic bomb survivors from the original data set constituted the data for this analysis. A convenience sample of Hiroshima atomic bomb survivors living in Tokyo or Hiroshima was recruited by Japanese researchers from a cross-cultural research team [34]. Data were gathered until saturation was achieved. From the original data set of 29 participants, those who described personal emergence in their story of surviving were included for this analysis. The data from 23 participants ranging in age from 75 to 88 years were considered in this analysis. 

Data collection/Preparation for analysis. The original study was noted as having human subjects’ approval in 2011 from the Tokyo Metropolitan Institute of Gerontology (TMIG). The participants were asked to describe their survival experience, according to a three-step process [35]: (1) the present health experience broadly described as “getting along day-by-day”; (2) the past health experience beginning with a description on 6 August 1945, up to the present; and (3) the future hopes for self and others. The time required for the interviews ranged from 20 to 60 min, and they were conducted at the survivors’ homes or in a private setting of their choice.

Participants’ stories were originally audio-recorded and transcribed in Japanese, then translated to English using a sequential forward-and-backward approach [34]. Both the Japanese and the English transcripts were considered in this secondary data content analysis process, beginning with the English transcripts and returning to the Japanese transcripts for specific points of clarification. All transcripts were de-identified with assigned numbers and reference to personal identifiers eliminated to assure confidentiality. In order to answer the research questions proposed for this analysis, the concepts of *personal emergence, connecting with earthy materials*, and *space-time expansion* were sought by reading and re-reading the original transcripts as previously described. Data analysis was supported with NVivo, a computer-assisted qualitative data analysis software (CAQDAS). 

Analysis. First, directed content analysis was conducted to identify story elements addressing Hiroshima survivors’ *personal emergence* and *connecting with earthy materials* (Table 1). This was done by extracting segments of contexts describing personal emergence (self-healing) using the software with the following search-words: healing, healed, healthy, better, well, etc. If the words were not identifiable, the personal emergence contexts were identified by rereading the interview transcripts with the personal emergence definition (Table 1) in mind. Once personal emergence segments were isolated in the transcripts, connection with earthy materials (nature components) became the focus. Finally, distance from the epicenter and time related to recognizing a sense of health, descriptors routinely addressed by survivors in the interviews, were identified for all participants. 

The empirical essence was heuristically synthesized into themes per concept within the theory of nature immersion (Table 1). At the final stage, *trustworthiness* was demonstrated for this analysis. Through peer-review and member checking the findings were discussed with the primary researchers who had extensive experience and familiarity with the data set. Cross-checking enabled consensual thematic categorization. 

## 3. Results

Characteristics of the 23 participants (Table 2), aged from 75 to 88 years old (*M* = 80; males (*n* = 13); females (*n* = 10)), indicated that they had personal emergence. The average age at the time of the atomic-bombing explosion was 19 years old with ages ranging from 14 to 27 years. The exposure to the atomic-bombing was categorized into two types: direct (primary) and indirect (secondary) hibakusha (a survivor of the atomic-bombing attack). One of the participants described himself as an indirect (secondary) hibakusha, explaining that, officially, the direct (primary) hibakusha had to be within the 2 km radius of the epicenter of the explosion. Twenty-six percent of the participants were considered a direct (primary) hibakusha within the 2-km radius and on average 0.98 km from the epicenter. Except for four participants who were more than 5 km away from the epicenter, the entire sample population of this study were within a 2.28-km-radius on average. 

Their health experiences had been ongoing from the time of the atomic bombing onwards (Table 2). Despite the ongoing or lifelong health challenges, the majority of the participants reported perceived health in general as “healthy” (83%) over “unhealthy” (13%) at the time of the interview.

### Qualitative Findings

Most participants had blast-related injuries: burns, skin-cuts, hard-blows, loss of consciousness, or blind eye(s). Thirteen percent of the survivors had no external injuries. However, those who had intact external bodies appeared to have silently deteriorated from the inside.

“*This one girl, she must have been freshman or sophomore at girls school, no injuries, no burns, she was well, and she just was munching away those rice balls, she probably ate 2 or more. Next morning when I woke up, she was laying all cold right next to where I slept. If I think about it now, it’s the radiation, you know? I should have asked her name and address at least, I couldn’t think that far. I feel bad.*”

Many indicated internal, besides external, radiation exposure. Some level of self-disorganization of internal systems was empirically manifested and described in the survivors’ self-reports: high-fevers, gastrointestinal symptoms (e.g., diarrhea, nausea, etc.), hepatitis, and nephritis. It took days to decades for the systems to self-organize the toxins, foreign bodies, or any residues from the blast traumas. For example, one survivor reported that 3 years after the blast trauma, pieces of glass that deeply stuck and embedded inside his leg had descended all the way down to the sole of his foot. The pieces finally got out through a small cut treated by the physician.

*They originally had been stuck up here in my leg, you could feel the bump right in there… I have less recovery power than normal people and it’d take much longer if I had a surgery. So I thought I’d rather not to have surgery if I absolutely don’t have to. I never thought it’d come all the way down to sole though.*

Another survivor’s story illuminated the manifestation of self-healing phenomena where healing force generated from within the self. The systems self-organized from within.

“*Doctors didn’t know what was causing (jaundice), no medicine, just eat well and take enough rest. I don’t remember exactly for how long but must have been like for a month or so, I had rested… Luckily jaundice had gone away maybe because I had been resting like I was told; I could function OK, didn’t have tiredness or heaviness of body*”.

There were scarce resources available in the aftermath of the fallout. “*There’s no medicine or anything*”, said the survivors. Self-healing appeared to be the only possible way for survival in the immediate aftermath of the bombing. 

While the internal nature of a person constantly attempted to re-harmonize or self-organize, external nature heralded a healing force. This was very opposed to what many survivors thought, “*There’s no trees, plants growing out from Hiroshima soil for at least another 80 years*”. Or “*There won’t be any trees and flowers growing back from Hiroshima soil at least for 75 years. Rumors then, all over the town…people who had been in Hiroshima got poison in their systems*”. The poisoned systems were both the internal and external nature alike. The first sprouting plant in the soil of Hiroshima in the fall of the year of the atomic-bombing attack was a surprise, which must have empowered the survivors to count on a synchrony of nature’s self-healing in humans. 

Earthy materials were sought after the fallout of the atomic bombing that annihilated nature as the city was thoroughly leveled: “*Nothing left, just the field all burned*”. One person even doubted the sight as “*I stepped outside of the station, and there was nothing, completely nothing, you know how they say desert on the moon*?” Another exclaimed, “*Then I looked, I saw a bridge, that bridge probably 2 km from the house you normally can’t see with all the houses and buildings, but I could see the bridge clear across. Oh, my god, it’s all destroyed*”. Onsite, there were almost no earthy materials left. Contacting with earthy materials that were unaffected from radiation appeared to be impossible. 

Nature immersion was nonetheless made possible. For those who evacuated from the city of Hiroshima to the countryside (e.g., mountains and farming fields), they often described how exactly they connected with earthy materials for self-healing. Others made whatever earthy materials left, useful onsite in the city. Personal emergence (self-healing) empirically manifested through connecting with earthy materials, while a sense of perceived health varied relative to space-time expansion factors. One extension of ideas that occurred in this analysis was the inclusion of human connection as a powerful force for healing (Table 3).

Many survivors expressed appreciation for a special person who provided occasions to connect with the nature of humanness. As earthy materials were scarce in the fallout environments, humans were the most available resources for connections. This was so common that it called for rethinking human-to-human connections as a sort of nature immersion. Humans’ oscillatory energies may be conceptualized as earthy expressions, like the heart and brain waves that exchange with each other during human–human engagement. Connections between humans may facilitate self-healing. For example, one survivor described appreciation for his father whose nature had reminded him of choosing to live rather than die:

*I tried to kill myself many times. But at the end, I pictured my father in my mind, then I couldn’t carry it out. What is going to happen if I died? My father, he had managed to live his life with his own hands, if I’d gone away, what would he think? When I thought about that, I couldn’t do it, I couldn’t carry it out. Many times I’d tried, committed to die, came this close…Born into unhappy society, I only had a father… had gone through so much to raise me. I grew up watching him trying so hard to raise me, every day watching my father work at it had given a lot of positives to my mental strength. If not for that, I wouldn’t have had a power to live through it then.*

Human emotions and thoughts could be considered energies of nature which emerge during connection between the two humans. Human-to-human connections were one of the major contributors for self-healing of the survivors because it generated “a power to live”. Continued narration from the man who talked about appreciating his father included the following passage:

*My father had said to me “it was good”. I thought “what? Good?…how can he say that? Well, he’s not the one who’s in this mess, loosing one eye and has injuries all over the body, of course you can say good” but he said, try to turn it around, think what would have happened if those injuries were deeper, what if you lost both eyes? It’s good, right?…he taught me that it’s all about how you look at things…I kind of got to thinking “you know, instead of loosing both eyes loosing sight completely, I can still see and that is a lucky thing if I think about it”.*

The human-to-human connections illuminated words of wisdom from the father to the son’s personal emergence. Human empathy connected the individuals as if waves of the two hearts resonated in rhythm, which could have generated the state of synchrony or coherence, the best functional mode for survival. Survivors appreciated this type of connection which was abundant during this time in Japan. “*Everybody is sympathetic and helping each other, that was a way of life, you know? Comparing what we have now, we say it was bad back then but might have been better that way*”. Survival and personal emergence were supported by human connections through which many hinted of healing traditions of old Japan, from the recipes of remedies to the ways of life. 

The beneficial human-to-human connections were more pronounced after the survivors started to share their own stories related to the atomic-bombings, which had long been buried deeply within themselves. There were fears of unknown impacts to the progeny from the unprecedented catastrophe of radioactive nuclear energies. “*So, all of this is nothing to do with my body. Those things (happening to children and grandchildren) are much more troubling. I didn’t write anything about my body and my health (in a copy of booklet that was shared with the interviewer), it’s all about my children*”. Despite the fears of disclosure to the public about the possible familial deficit, some later chose to pass on their own stories as a kataribe (storyteller). “*Around then I myself my body had been much better, I felt like, I thought that I finally could shake it (fears) off of me, I finally could break away from genbaku (atomic bombing), from radiation, I thought. I am worried that until when those kinds of things (illnesses of her children and grandchildren) would go on*”. Those kataribes experienced a sort of synchrony with other people, especially the young audience (junior or high-school students) who were sympathetic of human sufferings at war as well as peace in the present time: “*how precious and valuable the peace is now… and want to take parts in passing on what they learned*”. 

The heart-to-heart synchrony—the state of coherence—was felt by a kataribe as a power of own will: “*I have to keep passing on, I have to keep talking about it. That was how I started to talk*”. Another kataribe reported that his story opened the heart of the child audience. “*At the end, the child was listening to hibaku (being atomic bombed) story so seriously. The teacher writes me, ‘Student who was such trouble has changed so much after Hiroshima (stories)… this is a miracle.’ So now I know…story will get to his/her heart if you believe it would*”. The stories could be pervasive to the heart of the youngsters as a connection with the survivors, which generated personal emergence for both the listener and the survivor. “*This child started crying. My daughter, she goes, ‘Dad, the way you talk, it’s too real.’ I didn’t realize that…I feel like I can let people know about it*”. Some levels of the survivors’ woundedness started healing through these interactions.

In the traditional Japanese culture, earthy materials of nature include Mother Earth. Sounds of nature’s oscillatory energies reverberated deeply from Mother Earth and took the breath of the Hiroshima survivors on 6 August 1945.

*So we were all lined up and about to start (cleaning up the rubbles), then this Pika! (flash of bright light) came from my left and that earthshaking sound. Split second, can’t see anything, couldn’t breathe, thinking in my head “this must be death”. When things got calm, Hiroshima had already turned into ashes”*.

The shaking, shrieking, and screaming sounds of the ground were the very attestation of the wounded earth, which was parallel to the woundedness of the people. The feelings and sensations were real, and they altered the survivors’ psychoneuroimmunological (PNEI) systems. The survivors’ stories unraveled what once was intricately bound, manifesting in personal emergence, from their breathing to their healing.

From the qualitative findings, the main essence of the survivors’ self-healing appeared to be interwoven with nature, and human-to-human connections, which had long been spatially-temporally expansive and were now impacting survivors in the here-and-now as well as throughout the time from 6 August 1945 and into the future. 

## 4. Discussion

This study suggested that nature immersion was still possible between scarce earthy materials and human beings in the midst of wastelands of radioactive nuclear fallout in Hiroshima. Significantly, human–human connection rose in priority in the midst of earthy devastation. Earthy materials were rarely existent after the Hiroshima atomic bombing; both living and non-living systems from limestones to flaxseeds to humans were nearly annihilated but human connection flourished. 

The human–human connection appeared to be a must for survival in the aftermath of the manmade disasters. On the 20th anniversary of the 9/11 attack in New York City, the human connection was remarked as the upmost priority in the phenomena: “We became a family”, recalled by one of the organized groups of student survivors at New York University [36]. The university has the campus and dormitories uniquely located in downtown, close to ground zero. Their brief excerpts from the stories showed how they connected with others: called home, helped by connecting others with their families, or reconnected later because of the tragedy [36]. Even those who could not contact their loved ones made a family right where they were. One student asserted: “Though we came from different geographies and backgrounds, we chose to build upon what united us, in the midst of a world divided” [36]. The importance of connection with others was also reported by researchers who studied students distant from New York on 11 September 2001. Use of words indicative of connection as well as positive feelings increased from the morning to the evening of September 11 for these students [37] and there were slight shifts in cardiovascular indicators, such as blood pressure and heart rate. Overall, these students were reaching out to connect with others during this time of upheaval unlike anything they had previously experienced. The oscillatory nature of human rhythms, waves of the heart, brain, breath, were a dimension of human–human connection for the Hiroshima and the September 11 study participants. Although subtle, the synchrony of human rhythms for Hiroshima survivors may have had impetus for generating a healing force over the radioactive waves or any other residues traveling through the space. Natural space allowed for nature immersion to take in place, and it functioned as a vital precursor for healing force to sprout in this extreme environment.

Complexities of healing occurred in a nonlinear manner. The survivors questioned whether the human systems were poisoned. The answer to the question took lifelong twisted and tortuous healing paths. Some of those who had no external injuries had internal injuries, such as gastrointestinal disturbance that manifested in symptoms that came later and persisted for many years. Although chaotic at a glance, the capability of the human systems’ self-organization over years appeared to epitomize the natural laws that act the way the world works if the necessary conditions are met. Personal emergence or the generation of healing force is not random but occurs in the matrices of natural laws. The natural laws are universal for anyone and anything, when placed in the best possible condition to allow nature to act in the best way for healing to occur. The tenets of natural laws were raised in nursing by Florence Nightingale (1820–1910) who was adamant about empirically observing human health and healing as related to environmental conditions [38]. The synchrony of the rhythms of nature with that of humans might have been one of the conditions conducive to self-healing in the extreme environment, thereby meeting the tenets of natural laws.

Nature, as one of the survivors described, is best described with a Japanese word, *shinrabanshou* (森羅万象), which expresses not merely solid forms but free-flowing forms of micro-and-macrocosmic nature. Nature changes, cycles, or re-cycles in synchrony with ever-shifting seasons or rotations of the sun and moon. The notion of nature’s impulse can be attributed to animism or ‘*natural theology*’ [39]. Nature is able to transcend disharmony toward harmony again through new relationships or rhythms. Still, the mountains and rivers, as if accepting everything quietly, remain and heal themselves from within. New relationships or rhythms through connecting with earthy materials and other humans manifested in the survivors’ personal emergence, which can be translated into a word, *synchrony*, defined as a congruent feeling with another being [40] or environment. There are certain levels of *synchrony* between the survivors’ inner impulse and that of the plants. For instance, the two-dance or *biodance* [8] with nature, full of vulnerability as well as viability, was apparent for survivors as well as flora/fauna in the aftermath of the atomic bombing. Both the plant and the person are fragile as well as robust and resilient. The survivors may have seen the recuperating nature in themselves and their environments in unison. 

**Figure 1:**
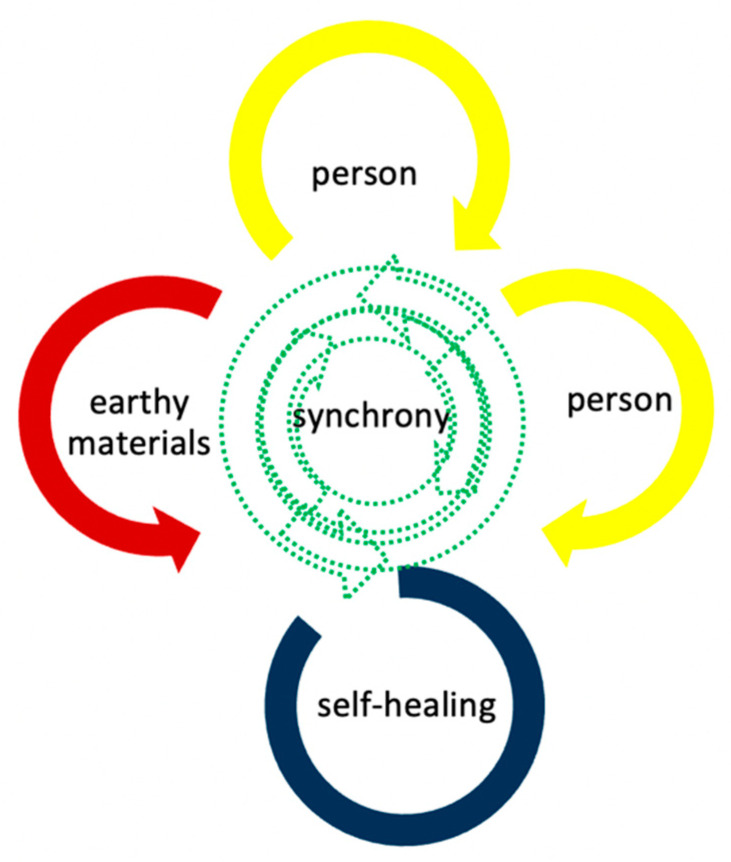
The nature immersion model for healing in the extreme environments.

Nature’s inner impulse, life force, or vital power is immanent in every element of nature, as small as flowers or as large as mountains. The fundamental building blocks of nature are often described as energy in quantum physics and spirit in theology. Energy or spirit can be both matter and non-matter, both particles and waves, and both living and non-living systems. Latent energy is inherent in nature’s fundamental building blocks, which may explain why there is a strong affinity or awe regarding nature (biophilia [4]) among humans. The affinity or awe regarding nature has traditionally been valued in the Japanese culture. When the fundamental building blocks of earthy materials are traumatized, connecting with earthy materials as well as other humans launches potential for synchrony and healing as depicted in the nature immersion model for healing in the extreme environment (Figure 1).

Personal emergence, an embodied healing force, generated from within the Hiroshima survivors, namely self-healing. Although the detailed contexts of self-healing varied among the survivors, connecting with earthy materials remained significant, as did connecting with other humans. Descriptions of varying sorts of synchrony occurring in connection with earthy materials or with other humans appeared to make a difference for survivors, and self-healing emerged from these connections. 

Implication for Research. Healing could occur without cure; the elimination of the signs and symptoms of diseases or injuries may or may not correspond to the end of the person’s illness [6]. The healing process is complex and multidimensional; it can be unstable and unpredictable in the systems of both natural space and humans. However, unstable nature allows the systems to catalyze healing shifts toward a new and higher order, and unpredictable nature allows the creativity of something new and deeper to emerge as a whole [41,42]. The contexts of natural space and human healing require a framework that flexibly encompasses the complexity science as well as spirituality. The multidirectional, not unidirectional, emergence of healing outside the predictable time-series frame unfolds through increasing complexities. An impulse and impetus of life-force for healing from within the person warrants further investigation that encompasses life change and challenge across generations and geographies.

Limitation. This study was conducted utilizing the existing narrative descriptions of Hiroshima survivors [34]. The original descriptions were not intended to be specific to understanding natures’ impact, as is addressed in the middle range theory of nature immersion. For instance, many parts of the descriptions of “getting along day-by-day” were unrelated to the theory concepts being explored, personal emergence, connecting with earthy materials, and space-time expansion. However, the data did allow for exploration of descriptions that were consistent with the theory concepts, resulting in new insights about the synchronies of person–earthy materials and human–human connections that guide thinking about the power of nature even in extreme conditions.

## 5. Conclusions

Little is known about human self-healing through contact with earthy materials in the midst of manmade catastrophic natural space, a gap that spearheaded this research study. There were earthy materials, intangible by the hands but tangible by the heart of the survivors immersed in a natural space infused with radioactive nuclear energy in Hiroshima. Likewise, there were rhythm–rhythm connections between humans. Oscillatory energies made synchrony possible, manifesting in healing at the levels of the cellular through consciousness. To the survivors, inner impulse, life-force, spirit, or divine power was immanent in both organic and inorganic systems of earthy materials and human connection. Immersing the self in ecological synchrony sprouted a seed of new relationships or rhythms designating these survivors as trailblazers in a journey of self-healing.

## Figures and Tables

**Table 1:** A summary of three core concepts in the nature immersion model [1] (pp. 388–389), [3].

Concept	Definition	Observable Quality
(a) Personal emergence	Healing that is an embodied force generating via self-organization of internal self-dimensions with external dimensions of nature to overcome disharmony and create harmony (e.g., among the cells to patch a wound together, transcending past and present memories to let go of emotions).	Self-healing
(b) Connecting with earthy materials	Sinking gently without intention to earthy constituents of nature including the organic and inorganic on Mother Earth, visible as the sunlight, invisible as sounds, tangible as stones, and intangible as scent. A personally developing relationship with any and all levels of the living and/or nonliving systems, physically and spiritually.	Nature components
(c) Space-time expansion	Spatial-temporal sensitivity and expansion beyond nature immersion occasions in the past impacting personal emergence in here-and-now and possibly throughout the cycles of life.	Distance from epicenter and time taken for sense of perceived health

**Table 2:** Characteristics of the survivors’ self-reported health experiences onwards from the Hiroshima A-bombing attack.

**Characteristics of the Participants and Self-Reported Health Experiences**	***%***	***M***	***SD***
**Age**		80	3.35
**Gender**			
Male	57		
Female	43		
**Age at the time of the A-bombing attack**		19	
**Distance in radius from the epicenter (km)**		30.6	13.38
Within the 5-km radius from the epicenter ^1^		2.28	1.2
**Perceived health in general at the time of the interview**			
Healthy	83		
Unhealthy	13		
Unanswered	4		
**Main trauma from the A-bombing attack**			
Burns	39		
Blast skin cuts with foreign bodies (e.g., pieces of glass)	26		
Blinded eye(s)	4		
A hard blow to the body	9		
Loss of consciousness	9		
No major external injuries	13		
**Health experiences within a few days or months after the A-bombing attack**			
Heart diseases (e.g., ischemic heart diseases)	22		
Fatigue	21		
Diarrhea	17		
High fever	13		
Blisters and bleeding	13		
Hair falling	9		
Jaundice	9		
Wound suppuration	4		
Nephritis	4		
**Ongoing or lifelong health experiences after a year from the A-bombing attack onwards**			
Cancer, cysts, tumors	25		
Hypertension	13		
Hematologic diseases	12		
Rheumatoid arthritis or inflammatory diseases	7		
Metabolic syndromes (diabetes, hyperlipidemia)	7		
Infectious diseases (e.g., tuberculosis, diphtheria)	7		
Lower back pain	4		
Spinal stenosis	4		
Meniere’s disease	4		

Note: *n* = 23 (male = 13, female = 10); ^1^ Cases (*n* = 18) within the 5-km-radius from the epicenter.

**Table 3:** A categorization matrix of nature immersion events of Hiroshima Atomic-bombing survivors.

	Nature Immersion Evidence		
(a) Personal Emergence	(b) Connecting with Earthy Materials		(c) Space-Time Expansion
Self-Healing; Healing Shift	Rhythm-Disorganization	Reorganization Effort	Distance from Epicenter and Time Taken for Sense of Perceived Health
“Healing cool on my burns”	Skin burns	Making ointment for burns from flaxseed oils and limestone powders (calcium carbonate) with zinc oxide at a factory. Treating others with burns in the same way as own burns.	2.3 km; a few decades
“The blisters that came up on our hands eventually went away”	Secondarily infection to the hands with blisters	Disinfecting gauzes in the boiled water and laying it out on the board to dry under the sun (as taking care of the injured people as a nursing student).	24.3 km; a few decades
“The injuries didn’t leave scars or keloids… and gradually disappeared”	Skin burns; easily-induced tiredness	Making cucumber juice on burns ("It was the old countryside remedy") and treating burns with ashes from funeral hall.	2 km; a few years
“It (a bleeding wound—a painless deep hole on the lower back) took a year to heal”; “I am healthier compared to days back”	A bleeding wound in a painless deep hole on the lower back	Heading to Funairihonmachi station from Funairihonmachi and further South to the fig farm at night of the bombing, while thinking about whereabouts of the brother and father. “We sat down there (a fig farm) for a moment, me, my younger sister and the neighbor’s baby”; having the wall clay (“filled in a deep hole in my wound”) washed off by the sister.	1.3 km; a few decades
“I gained my strength”	Symptomatic diarrhea and nausea	Arriving at western Miyajima by foot and then to hot spring sanatorium.	1 km; a few decades
“So in my case (tiredness and heaviness), things settled down within 2 or 3 years”	No major injuries; later diarrhea and tiredness	“I went to the mountain to get clay for him (military doctor at school), he’d mix oil paper and clay and vinegar. After that (some food distribution coming from military), we ran out of onions and pumpkins…”	1 km; a few decades

## Data Availability

No new data were created in this study. Data sharing is not applicable to this article.
